# Amoebic liver abscess – a cause of acute respiratory distress in an infant: a case report

**DOI:** 10.1186/1752-1947-3-46

**Published:** 2009-02-03

**Authors:** Mohammad M Saleem

**Affiliations:** 1 Department of Pediatric Surgery, Jordan University Hospital, PO Box 13546, Amman, 11942, Jordan

## Abstract

**Introduction:**

The usual presentation of amebic liver abscess in children is extremely variable and unpredictable. It presents with a picture of common pediatric illness that is fever, lethargy, and abdominal pain, and can go on to develop into a rare complication of rupture into the pleura to cause acute respiratory distress, which is another common pediatric illness. In our patient, diagnosis was not made or suspected in these two stages.

**Case presentation:**

This is the report of a 2-year-old male infant who presented with a 2-week history of anorexia, fever, and abdominal pain. A few hours after admission, he suddenly developed acute respiratory distress; chest X-ray demonstrated massive right pleural effusion that failed to response to tube thoracostomy. Limited thoracotomy revealed a ruptured amebic liver abscess through the right cupola of the diaphragm. The content of the abscess was evacuated from the pleural cavity, which was drained with two large chest tubes. Serological examination confirmed the diagnosis of ruptured amebic liver abscess. Postoperative treatment with antibiotics including metronidazole continued until full recovery.

**Conclusion:**

Diagnosis of such a rare disease requires a high degree of suspicion. In this patient, the diagnosis was only made postoperatively. The delay in presentation and the sudden onset of respiratory distress must be emphasized for all those physicians who care for children.

## Introduction

Amebic liver abscess is an uncommon disease entity, especially outside endemic areas. It is rare in young infants. We report the case of a 2-year-old infant that was missed as a case of liver abscess until it ruptured into the pleural cavity and caused respiratory distress. Diagnosis was made in retrospect. We suggest that this report of such a rare complication will be of interest to the common practitioner or pediatrician caring for children with fever and common gastroenterological conditions.

## Case presentation

**Figure 1:**
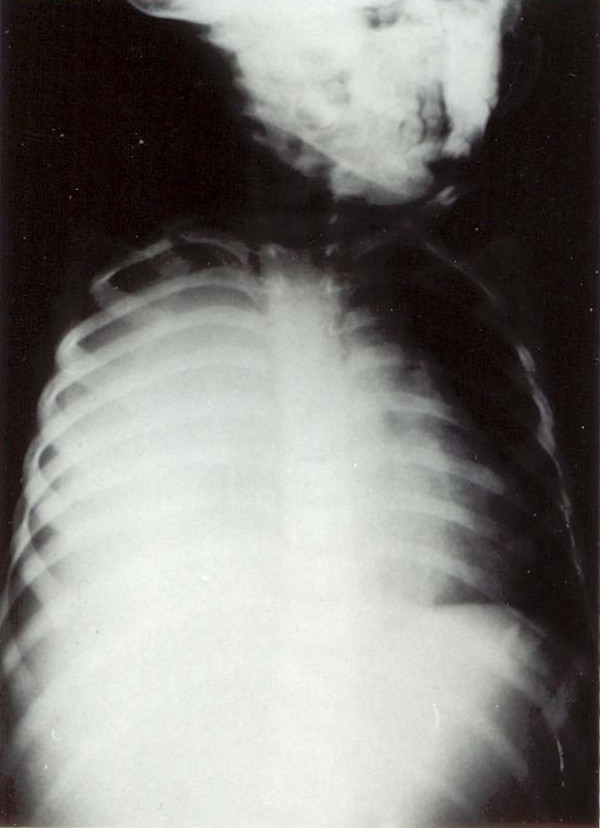
**Chest X-ray showing right-sided massive pleural effusion**. (A) Before drainage. (B) After drainage.

**Figure 2:**
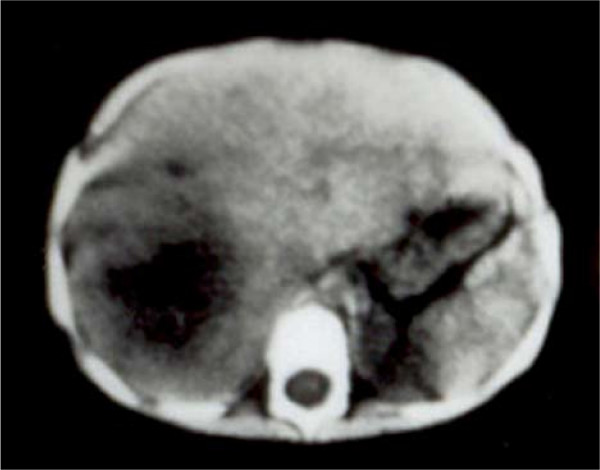
**Computed tomography scan of the liver showing the liver abscess**.

A 2-year-old infant presented to our hospital with a 2-week history of anorexia, malaise, fever and abdominal pain. He had been seen by several physicians who treated him for fever with antibiotics and antipyretics, without any improvement. On admission, his temperature was 38.9°C, pulse was 110/minute, and BP was 97/65 mmHg. Physical examination revealed a weak dehydrated infant, body weight 9.4 kg, < 50 percentile for his age. Abdominal examination showed tenderness of the right side of his abdomen, with an enlarged liver 5 cm below the costal margin. Auscultation of the chest was normal, and the rest of the physical examination was within normal limits. Laboratory investigations: Hb 9.5 g/dL, WBC 11.7 with normal differentiation, and normal platelets. Urine analysis and serum electrolytes were within normal limits. Upon admission, the infant was started on intravenous fluids. Empirical third generation cephalosporin was started. A few hours later, he developed a sudden onset of respiratory distress with severe tachypnea. Chest X-ray revealed opacification of the right hemithorax (Figure 1A). A chest tube was inserted; however, it failed to relieve the infant and did not yield any fluid. Only a small amount of thick membrane like material was obtained and this was sent for analysis and culture. Due to his continuing respiratory distress, the infant was taken to surgery where a right limited thoracotomy was performed. This revealed an obvious ruptured liver abscess into the chest. A thick cheesy material was evacuated and two large chest tubes were left to drain the chest cavity (Figure 1B). A rupture was seen through the cupola of the right diaphragm and thought to be an amebic liver abscess through the diaphragm into the chest. A computed tomography (CT) scan the following day revealed a large abscess cavity, shown in Figure 2. The patient was given metronidazole; in addition, serology of entamoeba and stool for amebae and parasites was ordered. The hemagglutination test was > 1:4000. The stool was negative for ova and parasites. A liver function test showed elevated alkaline phosphatase and mild elevation of liver enzymes. The infant was treated for 7 days parenterally, and oral metronidazole was continued for 6 weeks. His recovery was good. Follow-up chest X-ray after 6 months was normal. CT and ultrasound (US) follow-up of the liver at about 1 year showed the liver to be completely normal.

## Discussion

Amebiasis is endemic in the tropical and subtropical regions of the world [1]. However, amebic liver abscess (ALA) is rare, or is under-reported. Reports from western countries indicate a resurgence of amebic liver abscess associated with overcrowding, immigration, and reduced living standards [2]. It also contributes to public health problems in industrialized countries [3]. ALA develops in approximately 3% to 10% of patients who develop amebic infestation of the gastrointestinal tract (GIT). Complications of amebiasis as ALA are the most common manifestations of amebiasis outside the GIT [4,5]. It is more common in adults and is associated with more severe morbidity and mortality. Reports of ALA in children are sporadic [3-8].

The clinical presentation of ALA is extremely variable and unpredictable. The usual presentation in children is one of acute illness, with right upper quadrant pain, fever, and tender hepatomegaly. Our patient had a typical presentation but unfortunately was not diagnosed in the early stages. Diagnosis requires a high degree of suspicion, especially in young infants. A past history of dysentery is not common [9,10]. Our patient did not have a history of recent gastroenteritis. Leucocytosis, anemia, raised erythrocyte sedimentation rate (ESR), and alkaline phosphatase are common. Confirmation of the diagnosis therefore depends on serological tests and response to treatment. Jaundice is uncommon in children compared to adults. CT scan and US are useful in confirming the presence of the lesion, but cannot distinguish between pyogenic and amebic abscesses. Indications for needle aspiration of ALA are: no clinical improvement within 48 to 72 hours of treatment; abscess causing marked tenderness or severe pain; abscess > 10 cm in diameter; marked elevation of the diaphragm; most left lobe abscesses, and abscesses associated with negative serology tests [11].

Most amebic liver abscesses respond to medical treatment, and metronidazole is the amebicidal treatment of choice. A number of studies have been undertaken to define clearly the role of aspiration in the treatment of ALA [9-13]. Most concluded that routine aspiration of uncomplicated ALA is unnecessary [11], and individualized approaches to treatment should be preferred to routine percutaneous aspiration or surgical drainage. Rupture of the ALA into the peritoneal cavity occurs frequently in adults, but has only been seen in 1 in 24 children with ALA. Our patient recovered without surgical intervention. With aggressive aspiration of the abscesses, 1 in 48 ruptured into the pleural cavity, and a single one into the tracheobronchial tree requiring bronchoscopy [14]. Predisposing factors for the development of ALA are malnutrition and poor socioeconomic status, anemia, chicken pox, thalassemia, and teratology of Fallot [10,11]. Of the high mortality rates in earlier reports from South Africa and USA [15], 45% were due to contributing factors such as delay in diagnosis and the possibly relatively immature immune system in the very young child. Our patient had low body weight, anemia and belonged to a low socioeconomic class, otherwise he had no apparent risk factors.

## Conclusion

Diagnosis of such a rare disease requires a high degree of suspicion. In our patient, the diagnosis was only made postoperatively. The delay in presentation and the sudden onset of respiratory distress must be emphasized for all those physicians who care for children.

## Abbreviations

ALA: amebic liver abscess; GIT: gastrointestinal tract; CT: computed tomography; US: ultrasonography; ESR: erythrocyte sedimentation rate; WBC: white blood cell count.

## Consent

Written informed consent was obtained from the parents of the patient for publication of this case report and any accompanying images. A copy of the written consent is available for review by the Editor-in-Chief of this journal.

## Competing interests

The author declares that they have no competing interests.
